# New Clinical View on the Relationship Between the Diameter of the Deep Femoral Artery and Sex: Index δ-Anatomical and Radiological Study

**DOI:** 10.3390/biomedicines13061428

**Published:** 2025-06-10

**Authors:** Piotr Łabętowicz, Nicol Zielinska, Dawid Pilewski, Łukasz Olewnik, Kacper Ruzik

**Affiliations:** 1 Department of Forensic Medicine, Pathology and Histology, Mazovian Academy in Plock, 09-402 Plock, Poland; p.labetowicz@mazowiecka.edu.pl; 2 Department of Clinical Anatomy, Mazovian Academy in Plock, 09-402 Plock, Poland; n.zielinska@mazowiecka.edu.pl (N.Z.); k.ruzik@mazowiecka.edu.pl (K.R.); 3 Nursing Department, Mazovian Academy in Plock, 09-402 Plock, Poland; d.pilewski@mazowiecka.edu.pl

**Keywords:** deep femoral artery, vascular diameter, sex differences, anatomical variation, radiological anatomy, index δ, femoral artery morphometry, clinical anatomy, angiographic correlation, lower limb vasculature

## Abstract

**Background:** The femoral artery is a continuation of the external iliac artery. Knowledge of the topography and morphological variability of the thigh vessels informs various fields of medicine, such as hip replacement, hip fracture and femoral trochanter fracture, embolectomy, and angiography. The main aim of this study was to calculate the δ index from morphological measurements. We introduce the δ index to quantify the relative dominance of the DFA in supplying the thigh, aiming to improve clinical assessment and procedural planning. **Methods:** The study comprised two parts: anatomical dissection and radiological examination. During the anatomical study, 80 lower limbs (34 female and 46 male) fixed in 10% formalin were dissected. For the radiological study, angio-CT scans of the lower limbs of 100 patients (200 lower limbs) were analyzed. In both studies, the δ index was determined. This is the ratio of the diameter of the deep femoral artery at its point of origin to the diameter of the femoral artery after that origin. The morphometric measurements were analyzed statistically using Statistica 12.0 software. **Results:** The average values of the δ index for the right side were 0.95 (±0.23) and 0.89 (±0.21), respectively, in the anatomical and radiological studies, while for the left side they were 0.94 (±0.23) and 0.89 (±0.27), respectively. The average values for males were 0.88 (±0.18) and 0.80 (±0.17), respectively, while for females they were 1.04 (±0.26) and 1.12 (±0.23), respectively. **Conclusions:** The δ index, elaborated and calculated in anatomical and radiological studies, showed no statistically significant body side difference. However, it showed a statistically significant sex difference; there was a greater distribution of blood through the deep femoral artery in women than in men.

## 1. Introduction

The femoral artery (FA) is a continuation of the external iliac artery. Its main branch, the deep femoral artery (DFA), provides blood for the entire thigh area including the hip joint, the femur, and the anterior, medial and posterior groups of muscles [1,2,3].

Both its location of origin and the morphology of its branches are variable. The literature describes different distances of the origin of the DFA from the FA in relation to the middle part of the inguinal ligament. Usually, the DFA arises from the FA about 4 cm below the inguinal ligament [4]. However, some studies have measured this distance as 2.5–5.1 cm below the medial part of the inguinal ligament [5,6].

Knowledge of the morphological variations in vessels in this region is clinically significant, such as during revascularization, reconstructive, and orthopedic procedures [7]. Moreover, iatrogenic or traumatic injuries to the hip joint, which injure the vessels that vascularize it, can cause ischemia and, as a result, hip joint necrosis.

Importantly, DFA aneurysms can occur, and surgical procedures are still the gold standard for treating them. DFA aneurysms are not usually associated with any specific clinical symptoms so they are very difficult to diagnose. They have a high rupture rate [8].

Therefore, we propose the δ index as a novel morphometric ratio that reflects the relative contribution of the DFA to thigh perfusion. Unlike isolated diameter measurements, this proportional index accounts for interindividual size differences and offers a standardized way to evaluate femoral arterial dominance. It may serve as a useful parameter in planning vascular access, assessing bleeding risk, and tailoring surgical approaches in reconstructive and orthopedic procedures. The main aim of this study was to calculate the δ index (the ratio of the diameter of the DFA at its point of origin to the diameter of the FA after that origin) from morphological measurements. This index could be clinically significant for various clinicians, especially surgeons and orthopedists.

## 2. Material and Methods

Two studies were conducted. The first concerned anatomical examinations while the second concerned radiological examinations.

### 2.1. Anatomical Study

**Figure 1:**
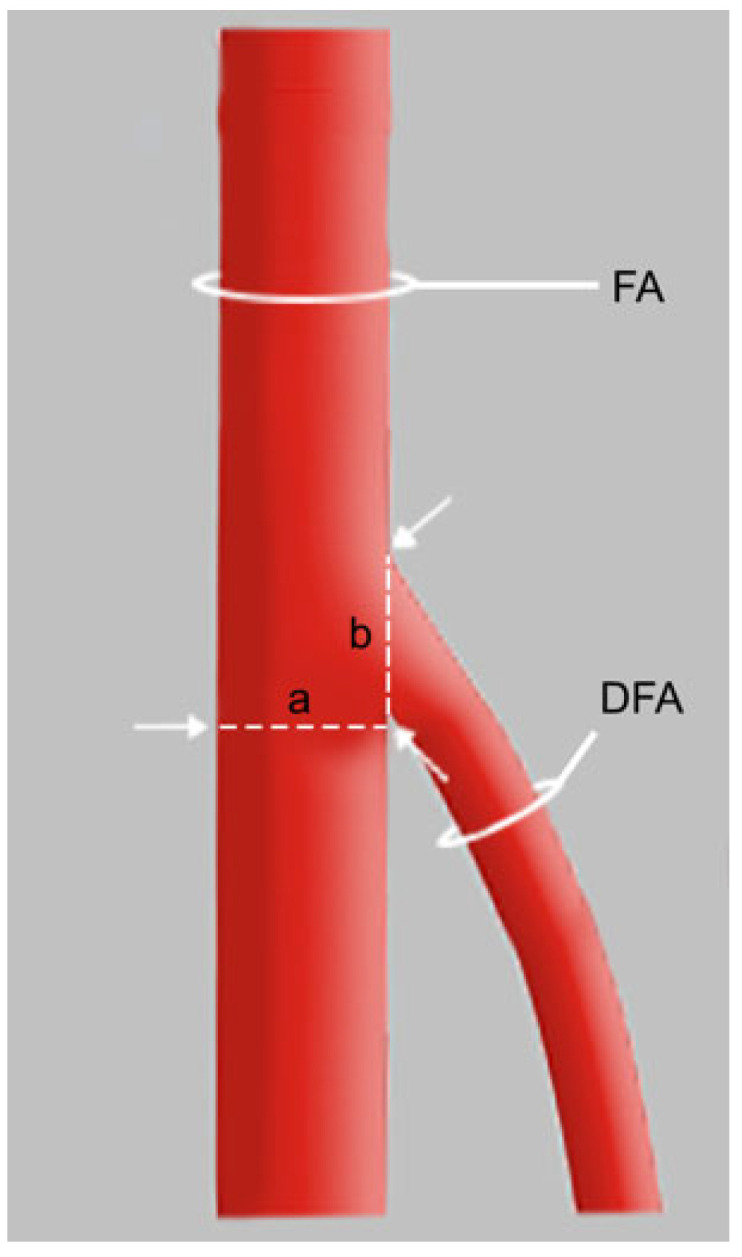
Schema of the measurements obtained. DFA—deep femoral artery, FA—femoral artery, a—diameter of the FA, b—diameter of the DFA.

Eighty formalin-fixed lower limbs were dissected (34 female and 46 male). The thigh region was approached, and the fat was removed. The femoral nerve and femoral vein were identified and separated from the FA. During the dissection, the morphologies of the FA and DFA were examined, particularly the branching patterns in the region of the femoral triangle. The DFA and its branches, the medial circumflex (MCFA) and lateral circumflex (LCFA) femoral arteries, were visualized and their branching pattern was noted. All measurements were performed using a calibrated Mitutoyo electronic caliper (±0.1 mm accuracy). The diameter of the DFA was measured at its point of origin, and the FA was measured 5 mm distal to the origin of the DFA. Two independent anatomists with over 5 years of experience performed all measurements. Each artery was measured by both investigators, and discrepancies greater than 0.2 mm were re-evaluated jointly. The final value used for analysis was the mean of the two concordant measurements. The δ index was defined and calculated. This index is the ratio of the diameter of the DFA at its point of origin to the diameter of the FA after that origin (Equation (1) and Figure 1).

(1) $$\delta =\frac{\emptyset \;DFA\;origin}{\emptyset \;FA\;after\;origin\;DFA}$$

Equation (1) shows the schema of the δ index, where FA—femoral artery, DFA—deep femoral artery, Ø—diameter, δ—index.

### 2.2. Radiological Study

The radiological dataset consisted of 100 anonymized angio-CT scans (200 lower limbs) obtained from patients undergoing routine vascular imaging unrelated to DFA pathology. The exclusion criteria included known peripheral arterial disease, femoral or iliac artery anomalies, and prior surgical interventions in the thigh region.

Scans were acquired using a 64-slice CT scanner (GE Healthcare, Milwaukee, WI, USA; 120 kV, 10 mA). Multiplanar reconstructions were analyzed using PACS software (https://postdicom.com/; GE Centricity, Chicago, IL, USA), and diameters were measured by two board-certified radiologists, each blinded to the other’s results.

Measurements were taken at anatomical reference points corresponding to those used in the anatomical study: the DFA was measured at its point of origin from the FA, and the FA was measured 5 mm distal to that origin. The mean of both measurements was used in the statistical analysis.

### 2.3. Statistical Analysis

Statistical analyses were conducted using Statistica 12.0 (StatSoft, Kraków, Poland). All continuous variables were expressed as mean ± standard deviation (SD). The Shapiro–Wilk test was used to assess the normality of distribution. Between-group comparisons (e.g., male vs. female, right vs. left limb) were conducted using Student’s *t*-test for normally distributed data and the Mann–Whitney U test was used for non-parametric data. A *p*-value < 0.05 was considered statistically significant.

## 3. Results

The δ index was determined from the morphometric measurements in both the anatomical and radiological studies.

Differences between body sides in the calculated mean δ index values were not statistically significant in either the anatomical or the radiological study: *p* = 0.8531 and *p* = 0.9480, respectively. The average values of this index for the right side were 0.95 (±0.23) and 0.89 (±0.21), respectively, while for the left side they were 0.94 (±0.23) and 0.89 (±0.27), respectively (Table 1).

However, there were statistically significant sex differences in the calculated mean values of the δ index in both the anatomical and radiological studies: *p* = 0.0031 and *p* = 0.0000, respectively. The average values for males were 0.88 (±0.18) and 0.80 (±0.17), respectively, while for females they were 1.04 (±0.26) and 1.12 (±0.23), respectively (Table 2). The calculated δ index shows that in females, the FA has a smaller post-origin diameter than the DFA itself at its point of departure from the FA. The opposite relationship is found in men.

## 4. Discussion

The primary contribution of this study is the development of the δ index, a novel morphometric parameter that quantifies the proportional diameter of the deep femoral artery (DFA) relative to the femoral artery (FA) at a standardized reference point. This index introduces a new dimension for assessing the vascular dominance in the proximal lower limb, particularly in light of significant interindividual variation and potential clinical consequences.

### 4.1. Embryological Background

Understanding the developmental basis of the femoral vascular system helps contextualize the anatomical findings. During embryogenesis, the sciatic artery—a branch of the internal iliac artery—initially supplies the lower limb. Around the eighth week of gestation, the femoral artery, derived from the external iliac artery, assumes this role [9]. Transient networks such as the rete femorale and rete pelvicum facilitate the remodeling of these arterial pathways [6,10]. Although rare, the persistence of the sciatic artery is still observed clinically and can complicate surgical access [11].

### 4.2. Anatomical Findings and Variability

The DFA is a critical branch of the FA, with considerable variability in its origin. Our findings are consistent with previous reports, indicating that the DFA most commonly arises 2.5 to 5.1 cm distal to the inguinal ligament [4,5,6,12,13]. In our anatomical cohort, the origin ranged from 28.85 mm to 78.24 mm, and in radiological assessments, it ranged from 44.43 mm to 60.35 mm. Moreover, we identified a statistically significant correlation between a larger FA diameter and greater DFA origin distance (*p* = 0.02). Such morphometric relationships are valuable during femoral triangle exposure.

Branching patterns also showed consistency with the literature. The DFA most frequently originated from the posterolateral surface of the FA in both anatomical and radiological cohorts (57.5% and 56.5%, respectively). The medial circumflex femoral artery (MCFA) and lateral circumflex femoral artery (LCFA) demonstrated typical emergence from medial and lateral surfaces, respectively [3,4,5,6]. These data further support the concept of predictable arterial corridors, despite the observed variation.

### 4.3. Clinical Implications of the δ Index

The δ index provides a standardized measure to assess the proportional contribution of the DFA to thigh perfusion. Our data reveal a clear sex-based difference: women exhibit a higher δ index, indicating greater relative blood distribution via the DFA compared to men. This has several clinical implications:

1. Surgical Planning: Greater DFA dominance in women may correlate with a higher risk of intraoperative bleeding during proximal thigh surgeries, including hip arthroplasty and tumor resections [14,15,16,17,18,19,20,21,22,23,24,25,26].
2. Flap Selection: In reconstructive surgery, particularly with anterolateral thigh (ALT) flaps, understanding local vascular dominance may influence flap viability and pedicle selection [27,28,29,30,31].
3. Access Site Management: Given the increased δ index in women, alternate vascular access routes (e.g., distal transradial) might be preferred for catheter-based interventions to minimize bleeding complications [32,33].

Additionally, the index may be useful in vascular mapping, aiding in the selection of graft materials or bypass routes in complex revascularization cases. In coronary artery bypass grafting (CABG), branches of the LCFA have already been employed successfully [34,35]. The δ index may support similar innovations by identifying robust, underutilized arterial conduits.

### 4.4. Broader Surgical Context

Our discussion also highlights relevant correlations with known procedural risks. For example, Stehli et al. Documented an increased bleeding risk in women following femoral access interventions, aligning with our findings [32]. Moreover, the anatomical information derived from the δ index could optimize outcomes in endovascular aortic aneurysm repair (EVAR), where anatomical suitability is paramount [36,37,38].

### 4.5. Future Directions

Future research should evaluate the prognostic utility of the δ index in larger, diverse populations. Potential studies could explore the correlations between high δ values and postoperative bleeding, flap failure, or graft occlusion. Machine learning models incorporating the δ index could also assist in preoperative planning. Such approaches could help personalize risk assessment and procedural strategies in vascular and orthopedic surgery.

## 5. Conclusions

The δ index offers a quantifiable anatomical metric with potential applications across vascular, orthopedic, and reconstructive surgery. Its sex-related variability emphasizes the need for individualized surgical strategies. By integrating this index into preoperative assessment, clinicians may enhance procedural safety and optimize outcomes.

## Figures and Tables

**Table 1:** Comparison of the δ index between the right and left lower limbs in the anatomical and radiological studies.

**The δ index** **vs.** **body side**	**Anatomical Study**				
	N—Right	N—Left	Mean (±SD) Value Right	Mean (±SD) Value Left	*p*-Value
	39	41	0.95 (±0.23)	0.94 (±0.23)	0.8531
	**Radiological Study**				
	N—Right	N—Left	Mean (±SD) Value Right	Mean (±SD) value left	*p*-Value
	100	100	0.89 (±0.21)	0.89 (±0.27)	0.9480

δ—delta index (ratio of DFA to FA diameter); SD—standard deviation.

**Table 2:** Comparison of the δ index between male and female subjects in the anatomical and radiological studies.

**The δ index** **vs.** **sex**	**Anatomical Study**				
	N—Male	N—Female	Mean (±SD) Value Male	Mean (±SD) Value Female	*p*-Value
	46	34	0.88 (±0.18)	1.04 (±0.26)	0.0031
	**Radiological Study**				
	N—Male	N—Female	Mean (±SD) Value Male	Mean (±SD) value female	*p*-Value
	142	58	0.80 (±0.17)	1.12 (±0.23)	0.0000

δ—delta index; SD—standard deviation.

## Data Availability

The original contributions presented in this study are included in the article. Further inquiries can be directed to the authors (Piotr Łabętowicz, Ph.D.–email address: p.labetowicz@mazowiecka.edu.pl).
